# Occupational Performance in the Discipline of Occupational Therapy During COVID-19 at a Public University in KwaZulu-Natal

**DOI:** 10.1155/oti/8801110

**Published:** 2025-01-22

**Authors:** N. Ndaba, P. Govender, D. Naidoo

**Affiliations:** Discipline of Occupational Therapy, School of Health Sciences, University of KwaZulu-Natal, Durban, South Africa

## Abstract

**Introduction:** The application of the person–environment–occupation–performance (PEOP) model in occupational therapy education offers valuable insights into the interaction between person, environment, and occupational factors and how these elements influenced the strategies used to adapt and navigate the transformation of health professions education during the COVID-19 pandemic. This study explored how individual characteristics, contextual demands, and the nature of educational occupations shaped the adaptation and performance of educators and students.

**Materials and Methods:** The study used a qualitative, exploratory research design at a public higher education institution (HEI) in South Africa, focusing on the occupational therapy program. Purposive sampling was used to recruit key participants (*n* = 44), including management, academic educators, clinical supervisors (academics), academic support educators, and occupational therapy students. Data collection occurred via the Zoom platform for 45–60-min semistructured interviews. Thematic analysis, guided by the PEOP model, was employed to analyze qualitative data, which was recorded, transcribed, and coded using NVivo (Version 12 Pro). The study ensured trustworthiness and adherence to ethical principles.

**Results:** The PEOP model was used as a framework to formulate the themes, namely, (1) person, (2) environment, (3) occupation, (4) occupational performance, and (5) occupational performance and participation. In the context of occupational therapy education, particular emphasis was placed on the adaptations and responses of educators and students as they navigated the demands of their educational occupations during the pandemic.

**Discussion:** The curriculum underwent rapid adjustments, requiring theoretical instruction and clinical practice modifications. These changes posed significant challenges and highlighted pre-existing gaps within HEIs, as experienced by educators and students.

**Conclusions:** This study highlighted the importance of supporting optimal occupational performance among educators and students, emphasizing the necessity of providing adequate resources and support. This ensures the well-being of educators and students, enabling effective engagement and participation as they navigate the evolving educational landscape.

## 1. Introduction

The COVID-19 pandemic disrupted and changed all sectors of society globally. Higher education institution (HEI) operations were particularly affected as measures to decrease the rate of infections curtailed in-person interactions [1]. The interruptions compelled HEIs to swiftly adapt, ensuring continuity in educational programs [2, 3]. As a result, many HEIs transitioned to online learning platforms and remote instruction, presenting unique challenges for educators and students in occupational therapy programs to maintain the effectiveness of their curriculum and ensure meaningful learning experiences. The COVID-19 pandemic particularly challenged occupational therapy educators in delivering effective education to students. Teaching methods and strategies had to be adapted to accommodate remote learning environments, integrating technology while considering contextual factors that influence student engagement and learning outcomes [1, 2]. Educators and students were required to rapidly change their methods of teaching, learning, and assessment to ensure optimal occupational performance outcomes within an evolving learning context.

Occupational performance and participation, as articulated within the person–environment–occupation–performance (PEOP) model [4, 5], encompass the dynamic interaction between an individual and meaningful occupations, framed by the person's intrinsic characteristics, the environment, and the inherent demands of the occupation. It is essential to recognize that occupational performance is not solely determined by an individual's abilities; rather, it is influenced by a multitude of factors, including environmental variables and occupational demands. These interconnected elements collectively shape a person's ability to engage in occupation, which ultimately has a profound impact on their overall well-being and quality of life. Thus, understanding and addressing these influences is critical for optimizing occupational outcomes and fostering meaningful participation [4, 6].

Occupational performance in the context of occupational therapy education in South Africa was significantly shaped by the transition to online and blended learning environments, reflecting the dynamic interaction between individuals, the environment, and the demands of the occupation. The person, comprising both educators and student, faced new demands as they adapted to the online learning model. The challenges they encountered were not only related to their personal abilities and adaptation but also the resources available in their environments. In HEIs with adequate technological infrastructure, educators and students were better able to engage in meaningful occupational tasks, such as teaching, learning, and assessment [7]. This positive interaction resulted in more favorable educational outcomes, where students' occupational performance in the learning process was enhanced through greater access to resources and support [8, 9]. However, in institutions with limited resources, the environment created significant barriers to occupational participation. Students faced difficulties in accessing essential materials, including reliable internet and learning resources, which compromised their ability to effectively engage in their studies [2, 8]. In response, both students and educators had to adapt by modifying their learning strategies, which involved creative solutions to bridge the technological gap. This required a shift in both the occupation, the tasks involved in teaching and learning, and the performance of those tasks, as educators needed to adopt new pedagogical approaches while students had to develop new skills and strategies to navigate the challenges of a less supportive environment [1, 10].

Moreover, the demands of the occupation, particularly the need for meaningful and engaging learning experiences, were met with resistance in fully online environments [11]. Studies revealed that students preferred blended or hybrid models, which allowed for more interaction and a sense of connection to their peers and instructors [8, 11]. The lack of face-to-face interaction in purely online settings often led to feelings of isolation, frustration, and reduced stimulation, factors that directly impacted occupational performance, as students struggled to remain motivated and engaged [11, 12]. These challenges emphasized the critical role of the environment in supporting occupational performance. In environments with limited social and mental health support, educators and students faced heightened difficulties, which impeded their ability to fully engage in the occupation of learning [13, 14].

Ultimately, this transformation in occupational therapy education demonstrated how occupational performance is deeply influenced by the interaction between personal characteristics, environmental factors, and the demands of the task at hand. The adaptation process that occurred within South African HEIs not only facilitated student learning but also shaped the development of pedagogical practices that fostered engagement, skill development, and a sense of competence. These changes in occupational performance reflect the evolving needs of students and educators, highlighting the importance of addressing the broader contextual factors that shape the ability to participate effectively in occupational therapy education. This study examined the perspectives and experiences of educators and students at a lower-resourced, public South African HEI offering a 4-year occupational therapy program accredited by the World Federation of Occupational Therapy (WFOT). The focus was on how the COVID-19 pandemic influenced their educational practices, challenges, and adaptations. The study highlights the disruptions caused by the pandemic and explores occupational performance and adaptation in response to these challenges using the framework of the PEOP model.

## 2. Materials and Methods

A qualitative, explorative research design was used for this study. The study was conducted at a public university offering a 4-year occupational therapy program in the KwaZulu-Natal province of South Africa during the COVID-19 pandemic. The program is offered within a health sciences school, offering seven other programs. Purposive sampling was used to recruit *n* = 44 key participants within the HEI. The participant groups included UKZN (University of KwaZulu-Natal) management representatives (*n* = 2), academic educators (*n* = 6), clinical supervisors (permanent academic educators that evaluate student clinical practice) (*n* = 8), academic support administrators (academic and clinical administrators) (*n* = 3), and occupational therapy students (*n* = 25).

Data collection occurred via an online Zoom platform, with 45–60-min semistructured in-depth interviews guided by peer-reviewed, literature-based interview guides for the data collection process. The relevant emerging literature from the pandemic period was reviewed by the authors, and key concepts were categorized to inform the development of the interview guides. The primary author and a research assistant conducted the interviews in English. A pilot study was conducted using five participants to test the data collection tool and identify any potential issues.

Qualitative data from the interviews were recorded, transcribed, and analyzed using a deductive approach to thematic analysis, with the PEOP model applied as the conceptual framework to identify and develop themes [15]. The initial data coding was completed using NVivo (Version 12 Pro) [16, 17]. The researchers were immersed in the data and reflected on biases and experiences throughout the process to enhance the credibility of the findings. The codes were grouped into similar categories and later into broader pre-empted themes using the PEOP model as a framework.

Trustworthiness was ensured through thick descriptions, investigator triangulation through regular meetings and reviews, and reflexivity to reduce bias [18, 19]. Credibility was ensured by purposively sampling participants, peer debriefing, and using verbatim quotes to support assertions. Transferability was ensured by reviewing the data multiple times. The study was granted ethical approval (reference HSSREC/00002508/2021). Each participant provided informed consent after perusing an information document prior to consent, and ethical principles of confidentiality and the right to withdraw were adhered to in this study.

## 3. Results

**Figure 1:**
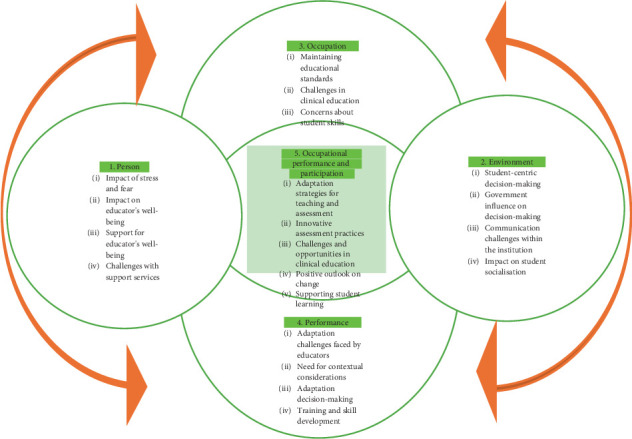
Summary of study themes using the PEOP model (adapted from [4]).

The PEOP model was used as the framework to formulate the themes (Figure 1).

### 3.1. Person

This theme addresses person factors, which refer to an individual's intrinsic characteristics, including physical, cognitive, and psychosocial aspects, that influenced occupational performance during the COVID-19 pandemic.

#### 3.1.1. Impact of Stress and Fear

Educators experienced elevated stress levels and had to adapt their teaching methods and strategies rapidly. This led to feeling overwhelmed, under pressure, and constant fear of the unknown virus. The work pressure caused by the pandemic contributed to their restlessness and unrelaxed states.

I think for me the first thing that comes when I think of my work as an academic and COVID-19, stress is the first thing that comes. It was so stressful to switch from different forms and strategies of teaching that we have been using and we have become so comfortable with them. (Academic educator, Participant 3)

#### 3.1.2. Impact on Educators' Well-Being

The emotional impact on educators due to the increased demands for mastery during the pandemic was prevalent. One educator expressed the emotional toll of providing personal contact information to students, leading to heightened anxiety and a shift to a 24/7 work mentality. This demonstrates the challenges faced by educators in maintaining boundaries and managing student expectations during the pandemic.

The mere fact of having to give students their personal number, I don't know if anyone would understand the impact it does emotionally on you because students are full of anxiety, they don't know that you work from eight to four- your hours of work have changed its twenty-four/seven now. Giving out that private information I just need to applaud the department and the academics following up on students, calling the students. (Academic support educator, Participant 6)

#### 3.1.3. Support for Educators' Well-Being

Educators expressed the need for additional psychosocial support to cope with the challenges brought about by COVID-19. The university was urged to provide additional assistance and support to ensure the well-being of educators. The importance of addressing the psychosocial needs of educators who were personally affected by the challenges brought about by the pandemic was raised.

The university must try its best to offer a lot of assistance and support to the educators as a whole. (Academic educator, Participant 5)

The university must try its best to offer a lot of assistance and support to the educators as a whole. I feel like more could be done in a sense that, you know, we could be looking at different avenues and offering additional psychosocial support for the staff on what COVID has meant to them and done to them and how COVID has shifted their teaching and their learning as well. (Academic educator, Participant 5)

#### 3.1.4. Challenges With Support Services

The challenges faced by students in accessing adequate support services were highlighted. Students mentioned the perception of support services being inadequate, with a student expressing frustration over the lack of appropriate therapy and resorting to medication due to the severity of their situation. This indicates a significant impact on students' mental health and well-being, showcasing the crucial role of support services in fostering a conducive learning environment.

The support service thing, it's a scam. I know getting the right type of therapist was a challenge, and I know people who were severely going through (stress) were hospitalised because of how their situation is. The support is there in theory, but it's not there. I went back on anti-depressants because I felt like I will not make it, if I don't have something to help me. (CSOT, participant FWV)

### 3.2. Environment

This theme focuses on environmental factors, which encompass the external context, including physical, social, cultural, and institutional influences, all of which impacted occupational performance during the COVID-19 pandemic.

#### 3.2.1. Learning Environment Challenges

The pandemic forced students to return to their homes, where their environments and socioeconomic backgrounds were not conducive to effective learning. This situation highlights the impact of home environments on students' educational experiences, underscoring the need for supportive learning environments beyond traditional campus settings. Students encountered difficulties when studying from home, lacking access to necessary resources and facing connectivity issues. The disparities in living environments affected students' ability to engage effectively in online learning.

We admit a lot of students from different socioeconomic backgrounds and different cultural backgrounds, as well as those that have struggled. For example, let's begin with technology. They have such a difficult time in navigating technology, in navigating online platforms, so the support in that, it has been numerous sort of tutorials that have been given by the IT department in terms of how to navigate Moodle or navigate online platforms. Additionally, data has been issued to the students by the university to assist the students in their homes and surroundings. Sometimes you find that they may still battle with their network, but bearing all of those factors in mind, as the department, we have had to make consideration[s] in the way students submit [assignments]. (Academic educator, Participant 3)

So those are the realities that face us; some of them are really beyond the university's control but these are the socioeconomic challenges that really face us, particularly us as UKZN we cater to the needs of those students we have a targeted agenda to attract and uh admit students from those backgrounds in order to give them an opportunity but these are the challenges that you know happened you know when we get students from those backgrounds it would have been much easier if those students were on campus where they have access to all the things particularly those who live in our common residences they could be in their own rooms they have opportunities to go to the libraries and use the University facilities use the LAN and all of those things but when they are home the reality is something else. (University management representative, Participant 1)

#### 3.2.2. Student-Centric Decision-Making

Significant communication challenges within the HEI management are evident. Decisions affecting educators and students were made solely from the managerial perspective, without considering the input of students or their representatives. This lack of involvement in decision-making processes directly impacted students in their unique contexts. The HEI management failed to acknowledge each student's individual circumstances and needs, leading to decisions being made on behalf of students without proper consideration of their home environments. Student input and representation were disregarded in decisions directly impacting them. This exclusion of students from the planning phase was evident, with higher-ups expressing reluctance to include student representatives. The absence of student voices in shaping decisions for their context and future was noted, indicating a disconnect between the institution and the student body.

I guess verbally, they would say they considered the students' perspective. I don't think the students were given enough voice in finding how things should be shaped within their context and for the future. (University management representative, Participant 2)

The feeling from higher up was that they don't want to include SRC at that level in the planning phase because they've made them derail the plans, but our experience as the institution is if you don't consult or consider, and that's not necessarily to say to give in to the students, but consider the voices of the students who are ultimately the beneficiaries- the people on the ground-who are mostly affected by these changes of rules if we don't consider their voice and their experiences we could be planning something that's not feasible. (University management representative, Participant 2)

#### 3.2.3. Government Influence on Decision-Making

The management responses were influenced by government directives, shaping the decisions made by the HEI. The decisions made by the HEI were directly influenced by government directives. This highlights the collaborative approach taken by the institution to align with external guidelines to manage the pandemic effectively.

I'm not a political person I'm not a political analyst; however maybe coming from you know a university that's funded, most of the resources are funded by the government, you can find that most of the decisions were from a government point of view not necessarily from an independent institution whereby they make their own decisions obviously still in respect and consideration of the government rules and regulations where policies are concerned. However there are certain things that you would expect for an internal independent investigation as to how the university can independently think will be best for the students. I didn't see a lot of that from the university, but mostly everything was dictated from a government point of view, and it was obviously important for the university to then respect that because in terms of the funding and support is concerned, but that did not necessarily have the best impact, for where the students and the staff is concerned. (Academic educator, Participant 4)

#### 3.2.4. Communication Challenges Within the Institution

There were challenges faced by the HEI management in terms of communication and clarity regarding the chain of command. This lack of clear communication led to confusion and poor accountability within the institution, affecting leadership effectiveness and decision-making processes.

I think structures were set up to help move things forward, but it wasn't really clear how these structures interact with each other, what is the sort of the chain in command, who the authoritative figures or the decision-making like entities within those structures, where does accountability lie for follow up? (University management representative, Participant 1)

#### 3.2.5. Impact on Student Socialization

The restrictions imposed during the pandemic directly impacted students' socialization opportunities. Due to social distancing measures, students experienced decreased time for physical socialization. This lack of social interaction could have affected their well-being and sense of community. The quote “students had decreased time for physical socialisation due to social distancing” highlights students' challenges in maintaining social connections.

Lack of social time did have some sort of effect on me because socialising is a crucial part of our biology, so yeah, not having physical contact with people or at least people that I have close relationships with. (UKZN student, Participant 4)

### 3.3. Occupation

This theme explores occupational factors in the context of educators and students, referring to the specific demands, roles, and responsibilities associated with teaching, learning, and assessment.

#### 3.3.1. Maintaining Educational Standards

The challenges faced in maintaining the integrity of health professions training at UKZN during the pandemic was highlighted. With limited access to hospitals and clinical settings, the institution had to redefine minimum competencies for health professionals in a disrupted environment. This highlights the necessity of adapting educational standards to ensure students are adequately prepared for their future practice.

So, the approach was to maintain the integrity of health professions training at UKZN and that's an ambitious statement in and of itself, because how do you do that in the context where most of our programmes are heavily clinical based. We didn't have access to hospitals, we didn't have access to patients even on campus, we didn't have access to appropriate online learning resources, so how do we maintain the integrity? The approach was opted to engage with regulatory authorities around what would be the minimum competencies, this isn't completely new, in a disrupted environment we can't hold students essentially to the same standard, how do we negotiate or redefine what we would consider to be minimum competences for health professionals, specifically those who need to complete in the academic year and go out to practice. (University management representative, Participant 1)

#### 3.3.2. Challenges in Clinical Education

Regarding clinical education, educators found that students struggled to integrate theory into clinical skills, compromising the quality of clinical education. There was a lack of hands-on input, and the demonstrated clinical skills did not meet the appropriate standard. Educators emphasized the need for more exposure to real-life clinical placements and experiences, as students were not receiving adequate training and exposure to clinical scenarios during the pandemic.

We didn't get a lot of experience with different diagnoses with different conditions… purely because we didn't see a lot of patients… there weren't a lot of patients at the hospital at that time. (CSOT, participant FWP)

In all this time, we had this constant worry that we were losing academic time, our students were losing clinical hours… they weren't going to get the hours that they need to graduate. (Academic supervisor, participant FWC)

#### 3.3.3. Concerns About Student Skills

Furthermore, educators raised concerns about the level of clinical reasoning, assessment, and intervention skills of students. They identified gaps in students' required skills and highlighted evidence of immaturity and incompetence in clinical and professional skills. This raised questions about the occupational therapy curriculum's ability to develop these skills in students during the pandemic.

I cringe again because for me I see a huge gap in the expectations of what the third year student is supposed to present with in terms of clinical reasoning and analysis and just the integration of theory into practical skills. There is a huge clinical gap from the second year to the third year, and unfortunately, I foresee a huge lack of skills in assessments and interventions, especially in the theory and the clinical aspects in the fourth year. This actually makes me wonder what kind of students we will be producing in a community level whereby they will have to go out and serve the community in 2023. (Academic educator, Participant 4)

### 3.4. Performance

This theme focuses on occupational performance, which refers to how educators and students engaged in and executed their roles and tasks within the context of the pandemic. It examines how their ability to perform educational activities was influenced by the interaction of person, environment, and occupation during this challenging period.

#### 3.4.1. Adaptation Challenges Faced by Educators

Educators faced significant challenges adapting to remote teaching and learning methods during the pandemic. The sudden need to change teaching strategies and assessment methods created high stress among educators. As one participant mentioned, “educators had to rapidly change the methods and strategies used for teaching, learning and assessment during the pandemic.” This rapid shift in teaching approaches led to feelings of being overwhelmed and pressure among educators.

#### 3.4.2. Need for Contextual Consideration

The necessity of considering the diverse contexts in which students are situated when implementing learning strategies was emphasized. It criticizes the approach adopted by the HEI management, pointing out that students' environments play a crucial role in their ability to engage with academic content. The importance of moving away from a “one size fits all” approach in education recognizing students' diverse backgrounds and challenges was highlighted. It acknowledges the necessity of considering individual circumstances, such as connectivity issues in rural areas and language barriers. This highlights a need to adapt educational strategies to cater to the unique needs of each student in a more individualized approach that considers each student's unique needs and contexts to ensure effective learning outcomes.

There was a “one size fits all” approach and we can't have a “one size fits all” approach, you know some students live in urban areas at home in privileged environments, others live in urban areas but not so privileged environments, but they have reasonable connectivity, those in rural areas have connectivity challenges, electricity challenges. (University management representative, Participant 2)

#### 3.4.3. Adaptation Decision-Making

The challenges faced in decision-making processes within HEIs regarding remote teaching and learning were highlighted. It is evident that the HEI management made decisions without considering the unique circumstances of each student.

They used a blanket of approach which again led to some inequities because even though data was being allocated, if the students are sitting somewhere in the rural areas, then data has no use to them because connectivity is a challenge. (University management representative, Participant 2)

#### 3.4.4. Training and Skill Development

In response to the pandemic, the university conducted extensive training sessions for educators to adapt to the new online teaching environment. Educators were trained in various aspects, such as setting up quizzes, online tests, and creating videos. This training was crucial in adapting to the new occupational demands in an online teaching environment, showcasing the institution's efforts to support educators in enhancing their technological skills for effective teaching during the pandemic. An educator reported, “the university did extensive training with the educators, how to set up a quiz, how to set an online test, how to make a video.” This proactive approach by management facilitated the transition to online teaching and learning.

The university did extensive training with the educators, how to set up a quiz, how to set an online test, how to make a video. (Academic supervisor, participant FW)

The university handled things very well… they just went in provided the training. It was happening in real time as you need the skill. (Academic supervisor, participant FWM)

### 3.5. Occupational Performance and Participation

This theme addresses occupational performance and participation, emphasizing how educators and students adapted their engagement in teaching and learning during the COVID-19 pandemic. It highlights how these adaptations enabled continued participation in meaningful educational activities despite the disruptions caused by the pandemic.

#### 3.5.1. Adaptation Strategies for Teaching and Assessment

In response to the challenges of remote teaching, educators had to adapt their teaching methods by redoing lectures and utilizing voice-over PowerPoint presentations. Additionally, academics had to rethink assessment practices, considering tests and exams as open-book due to the online nature of assessments. These adaptations reflect the need for innovative approaches to ensure effective teaching and assessment in a remote learning environment. The adaptation is required in teaching methods to accommodate the changing educational landscape. Educators had to extend their roles to provide extra support and mentorship to ensure students could effectively grasp the knowledge presented. This adaptation reflects a proactive approach to addressing the evolving needs of education.

You know, I think, like I said before, health sciences has tried its best. At first, I would have assumed that they needed to explore various mediums and various platforms as to how they can add clinical components to the students' distance learning, but they honestly tried their best. However, there are not a lot of online platforms that allow for clinical exploration and clinical learning. (Academic educator, Participant 4)

I mean we had to re-do all our lecture notes and all our PowerPoint and stuff because those files are just so massive so we really, really had to be cognisant on space and size, especially when doing a voice over on it as well. I still don't love it at all, but in any case, it's done for now until we find a better [way] around those things. You have to break an hour's lecture into like four parts; otherwise, that file is just so massive, and it takes so much data to download, or you can't email it. (Academic educator, Participant 3)

#### 3.5.2. Innovative Assessment Practices

The innovative assessment practices adopted during the pandemic were an adaptive response for educators as they had to transform their assessment practices to meet the needs of the program. Academics had to reconsider traditional assessment methods and shift toward open-book exams due to the online nature of education. This change was aimed at ensuring the credibility of assessments while acknowledging the students having accessibility to their notes. However, the shift in assessment practices showcases a willingness to adapt to new educational challenges.

In terms of assessment and even to do exams online, I was also thinking whether my assessment is really credible- the answers that the student is writing, is this what the student knows or [does] the student [have] a piece of paper around referring to it?. We do hide things on the learning sites but you can simply refer to the notes and answer everything. (Academic educator, Participant 1)

We need software, some sort of stricter measures in administering online assessments, but again, it's a year later and now we may be starting to see the light at the end of the tunnel, including some of those tools. (University management representative, Participant 1)

#### 3.5.3. Challenges and Opportunities in Clinical Education

There were challenges and opportunities in clinical education strategies during the pandemic. Alternative methods like Telehealth were embraced to continue clinical training. However, students felt underprepared and overwhelmed in clinical practice due to limited practical experience. This highlights the importance of balancing technological advancements with hands-on training to adequately prepare students for real-world scenarios.

Alternative methods for clinical education training were used, such as Telehealth during the pandemic. (Academic supervisor, participant FWC)

#### 3.5.4. Positive Outlook on Change

Despite the challenges faced in transitioning to online teaching and assessment methods, there is a recognition of the positive aspects of change. The benefits of exploring new ways of teaching, conducting assessments online, and engaging with students through digital platforms were highlighted. This positive outlook reflects embracing innovative approaches to education.

But while it was frustrating, there were also some positive things in the change as well; I saw that actually there is a benefit in doing things differently in a new way, using online teaching, using assessment of students online, doing presentations online, and engaging students on zoom and listening to them. (Academic educator, Participant 1)

#### 3.5.5. Supporting Student Learning

Furthermore, the participants highlighted the commitment of educators to support student learning and engagement of content. By offering additional tutorials and assistance, particularly in bridging language barriers, educators demonstrated a desire for upskilling to ensure that students have the necessary support to excel in their studies.

The university did extensive training with the educators, how to set up a quiz, how to set an online test, how to make a video. (Academic supervisor, participant FWR)

## 4. Discussion

The COVID-19 pandemic forced educators and students in occupational therapy programs to respond and rapidly adapt to unprecedented changes and challenges. This study explored the adaptation process within a South African HEI, exploring how individuals, namely, educators and students, navigated pandemic-induced disruptions and, more specifically, how these individuals adjusted to their environments and the various factors influencing their occupational performance and participation in the occupational therapy education program. Occupational performance is influenced by many factors and is essential for enhancing quality of life [4, 5]. It involves a transformative response to overcoming challenges stemming from the interaction between the person and their environment during meaningful occupation [4]. The pursuit of occupational performance, engagement, and participation during this period was driven by personal aspirations and contextual demands, shaping new blended pedagogical approaches in occupational therapy education.

The onset of the pandemic introduced heightened expectations across teaching, learning, assessment, and clinical education, facilitating innovation needed to develop new blended pedagogical approaches in occupational therapy education [2]. Psychosocial barriers, such as fear, stress, and mental health challenges, often exacerbated by inadequate institutional support, significantly impacted students' learning and interactions with educators [8]. Educators and students demonstrated occupational resilience, an ability to adapt to, transform, and negotiate occupations in the face of stressful and dynamic environments. This capacity is essential not only for individual well-being but also for maintaining functional effectiveness in challenging contexts. The ability to navigate and adjust occupational roles in response to stress is fundamental for sustaining productivity and well-being; therefore, fostering occupational resilience should be prioritized in educational and professional environments, as it enables individuals to thrive despite adversity [20, 21].

Furthermore, educators grappled with challenges in fostering competent graduates, meeting module objectives, and effectively participating in remote teaching platforms. The uncertainty surrounding students' preparedness and competence directly challenged the perceived quality of education and graduates produced [22]. The study findings align with global literature reporting heightened emotions; adverse mental health outcomes; and concerns about training standards, student engagement, and educator effectiveness. Globally, occupational therapy education programs experienced similar emotional and professional uncertainties, with concerns about maintaining training standards during remote learning. However, amidst these challenges, HEIs played a pivotal role in supporting both educators and students through skills training and development initiatives [11, 12, 23]. Institutional leadership was instrumental in cultivating an environment that nurtures the development of educators' and students' skills and well-being to enable participation in educational programs [24]. Educators and students within this environment relied significantly on the support. By providing robust infrastructure and student development initiatives, HEIs enabled participants to navigate the educational transformation effectively [13].

The COVID-19 pandemic precipitated rapid and profound changes in the educational landscape, driven by governmental directives and decisions affecting HEIs. Amidst these changes, educators and students encountered significant, revealing systemic challenges, including social isolation, inadequate support systems, and resource deficiencies in essential physical resources such as electronic equipment and network connectivity [2, 8]. These obstacles underscored the importance of tailored support mechanisms. Hence, HEIs must meticulously consider and address the specific, individualized environmental and contextual influences when adapting to new or evolving learning environments [25]. This is particularly relevant as graduates emerging from HEIs represent diverse backgrounds, each contributing to their unique educational journey. Low-resourced HEIs must prioritize the needs of their students, considering their socioeconomic and contextual realities when adapting or evolving learning environments. This is essential, particularly for institutions serving diverse student populations [2, 26]. In South Africa, the Council for Higher Education's (CHE) shift from permitting only face-to-face instruction at traditional universities to embracing blended learning marks a crucial evolution in the higher education landscape. This change is not merely a response to the pandemic but a necessary adjustment to accommodate the significant advancements in teaching and learning that were achieved during the COVID-19 era. The rapid adaptation to online and hybrid formats demonstrated that education could continue effectively in diverse and flexible formats, challenging the traditional reliance on in-person teaching. Therefore, this regulatory shift acknowledges the need to integrate these innovations into the long-term framework of higher education, ensuring that institutions remain resilient, accessible, and responsive to the evolving needs of students in an increasingly digital world (CHE, 2024).

Ultimately, the pandemic highlighted that the dynamic interplay between individuals and their environment must be adequately supported to meet the demands of developing a graduate who is capable of providing a relevant occupational therapy service while delivering the curriculum using blended learning. It demonstrated the necessity for adaptive learning strategies that equip educators and students with the skills to thrive in evolving pedagogical contexts. This meant reimagining clinical training, support structures, and teaching and learning methodologies for occupational therapy education to ensure continued professional development [27].

## 5. Conclusion

The study of occupational therapy education during the COVID-19 pandemic, viewed through the lens of the PEOP model, highlights the complex interplay between person, environment, and occupational factors in shaping educators' and students' adaptation to the challenges posed by the pandemic. The findings emphasize the critical importance of institutional support in fostering an environment conducive to occupational performance and participation. Robust support systems, including access to resources, mental health support, and adaptable learning strategies, are essential for enabling educators and students to successfully navigate disruptions and maintain meaningful participation in their educational programs [1, 21].

Ultimately, the success of adaptation is not solely dependent on personal characteristics but also on the environmental systems and institutional infrastructure that enable educators and students to perform and participate effectively. HEIs must address the diverse needs of their educators and students by providing tailored support mechanisms that promote inclusivity, participation, and engagement, ensuring that all participants can thrive in the evolving educational landscape [21, 28].

## Data Availability

The data that emerged from this study has been analyzed and reported in this paper. It can be accessed from the corresponding author on reasonable request.
